# Hematological trajectories and pregnancy complications in essential thrombocythemia and polycythemia vera

**DOI:** 10.1007/s00277-026-07236-1

**Published:** 2026-08-13

**Authors:** Freja Rueholm Klok, Finn Stener Jørgensen, Christina Gallø Schioldann Nielsen, Morten Kranker Larsen, Morten Dahl, Vibe Skov, Lasse Kjær, Christina Ellervik, Hans Carl Hasselbalch, Henrik Løvendahl Jørgensen

**Affiliations:** 1 Department of Clinical Medicine, Faculty of Health and Medical Sciences, University of Copenhagen, Copenhagen, Denmark; 2 Department of Clinical Biochemistry, Copenhagen University Hospital Hvidovre, Kettegård Alle 30, Hvidovre, DK-2650 Denmark; 3 Fetal Medicine Unit, Department of Obstetrics and Gynecology, Copenhagen University Hospital Hvidovre, Hvidovre, Denmark; 4 Department of Hematology, Zealand University Hospital, Roskilde, Denmark; 5 Department of Clinical Biochemistry, Zealand University Hospital, Køge, Denmark; 6 Department of Laboratory Medicine, Boston Children’s Hospital, Harvard Medical School, Boston, MA USA

**Keywords:** Essential thrombocythemia, Polycythemia vera, Myeloproliferative neoplasms, Pregnancy, Preeclampsia, Platelet counts

## Abstract

Women with essential thrombocythemia (ET) and polycythemia vera (PV) are at increased risk of pregnancy complications. During pregnancy, hematologic parameters tend to approach normal values, and previous studies have reported that a greater decline in platelet counts in ET is associated with reduced risk of complications. In this study, we examined longitudinal trajectories of platelet count, hemoglobin, hematocrit, and leukocyte count during pregnancy, characterized pregnancy outcomes, and investigated the association between platelet trajectories and preeclampsia. We established a longitudinal cohort of pregnant women with ET and PV in the Capital Region and Region Zealand between 2010 and 2024, using data from regional laboratory information systems and Danish health registries. In total, 41 ET and 5 PV pregnancies in 35 women were included. The mean age at MPN diagnosis was 30.1 years in women with ET and 34.1 years in women with PV. In ET pregnancies, platelet counts, hemoglobin, hematocrit and leukocyte count changed by − 44%, − 5%, − 12% and + 55%, respectively, from baseline to delivery, and in PV pregnancies − 36%, − 26%, − 25% and + 47%, respectively. Overall, 29% of ET pregnancies had a maternal complication, with preeclampsia being the most common (12%). ET women who developed preeclampsia had significantly higher platelet counts before pregnancy and experienced a greater decline in platelet counts during pregnancy than women without preeclampsia. Thus, hematologic trajectories during pregnancy may help identify women at increased risk of preeclampsia.

## Introduction

Essential thrombocythemia (ET) and polycythemia vera (PV) are chronic Philadelphia chromosome-negative myeloproliferative neoplasms (MPNs) characterized primarily by elevated platelet count and elevated hematocrit, respectively [1, 2]. Although MPNs are most frequently diagnosed in older adults, up to 20% of all patients are diagnosed during the reproductive years [3–5]. In contrast to PV, ET is typically diagnosed at a younger age and occurs more frequently in women, making ET pregnancies more common than PV [6]. MPN pregnancies are rare and occur in approximately 3.2–12.2 per 100.000 pregnancies annually [5, 7]. However, MPNs are likely underdiagnosed in the general population [8], and together with increasing maternal age and improved screening strategies, this may lead to earlier diagnosis and an increasing number of pregnancies complicated by MPN [9]. Hematologic parameters approach normal values during pregnancy, but women with ET and PV are still at increased risk of thromboembolic, maternal, and fetal complications during pregnancy [5, 9, 10]. Previous studies report that a greater decline in platelet counts during pregnancy is associated with decreased risk of pregnancy complications in women with ET, indicating that platelet trajectories may have prognostic value in ET pregnancies [10].

In this cohort of 46 pregnancies in women with ET and PV, the primary aim was to characterize pregnancy complications and examine longitudinal trajectories of routine hematological parameters – including platelet count, hemoglobin, hematocrit, and leukocyte count – from three months before conception to three months postpartum. The secondary aim was to investigate the association between platelet count trajectories and preeclampsia.

## Materials and methods

### Study design and data sources

This study included pregnant women with ET and PV from the MPN-CopZea-Repro Cohort, a register-based cohort from the Copenhagen Capital Region and Region Zealand, Denmark, investigating reproductive health in Danish MPN patients. We included women with a recorded first-trimester screening between 2010 and 2024. More than 90% of pregnant women accept the combined first-trimester screening in Denmark, and as the underlying cohort of pregnant women was not known prior to laboratory data extraction, we used this screening as a proxy for pregnancy [11]. For the included women, we extracted all available routine laboratory data from 2010 to 2024 from the laboratory information systems in the Capital Region (Labka II) and Region Zealand (BCC). These data were uploaded to Statistics Denmark and linked to the Danish National Patient Registry (NPR) and the Danish Medical Birth Registry (MBR) using unique personal identifiers to receive information about diagnoses and pregnancy outcomes. Data were accessed via secure remote servers in pseudonymized form.

### Study population

**Fig. 1:**
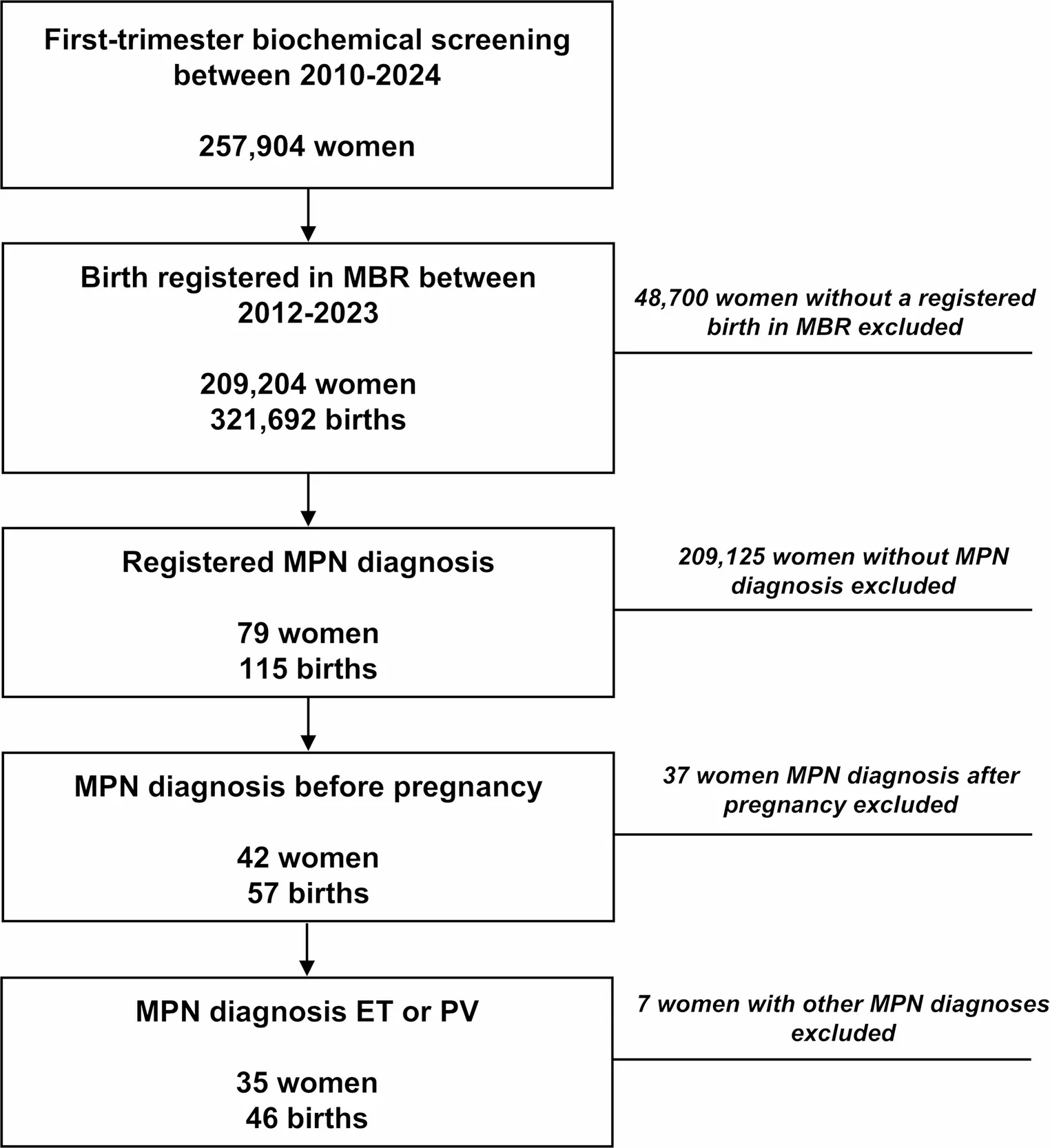
Flowchart of the selection of study subjects.*ET* essential thrombocythemia, *MBR* medical birth registry, *MPN* myeloproliferative neoplasm, *PV* polycythemia vera

The base cohort comprised 257,904 women aged 18–50 years with a baseline first-trimester biochemical screening in the Copenhagen Capital Region and Region Zealand, Denmark, between 2010 and 2024 (Fig. 1). Only women with a registered pregnancy in MBR between 2012 and 2023 (209,204 women and 321,692 births) and a registered MPN diagnosis in NPR between 2005 and 2023 (79 women and 115 births) were included. We excluded women diagnosed with MPN during or after pregnancy (37 women) and only included women with ET or PV (35 women and 46 births).

### Routine laboratory measurements and MPN diagnoses

Routine laboratory measurements (platelet count, hemoglobin, hematocrit, leukocyte count) were extracted from the regional laboratory information systems. All analyses were performed by hospital laboratories located in the Capital Region and Region Zealand, Denmark. For each pregnancy, we included all available blood test results from three months before conception to three months postpartum. MPN diagnoses were identified from the NPR for the period 2005–2023. We included the following ICD-10 codes: ET (D47.3, D75.2), PV (D45), primary myelofibrosis (PMF) (D47.4 A), secondary MF (D47.4B–C), and MPN unspecified (MPN-U) (D47.1). Due to Danish data protection regulations, categories with fewer than five must not be reported, and the cohort was consequently restricted to women with ET or PV.

### Covariates

Information on maternal characteristics and pregnancy-related factors was obtained from the MBR, including maternal age, maternal body mass index (BMI), smoking status, fetal birth weight, placental weight, fetal gender, and mode of delivery. Smoking status was categorized as smoking during pregnancy or not. Birth weight z-scores were calculated using the Fenton 2013 reference [12]. Small-for-gestational-age was defined as birth weight below the 10th percentile for gestational age and sex, and large-for-gestational-age was defined as birth weight above the 95th percentile [12]. Preterm delivery was defined as delivery before gestational week 37. Pre-existing or pregnancy-related diagnoses, including gestational hypertension (O13), preeclampsia (O14, O15), gestational diabetes mellitus (O244), intrahepatic cholestasis of pregnancy (O266G), pregnancy-related bleeding (O20, O46, O67, O72), thromboembolic events (I21, I23, I26, I63, I74, I81, I82), diabetes (E10-E14), and hypertension (I10-13, I15) were retrieved from the NPR.

### Statistical analyses

Descriptive statistics were reported as mean and standard deviation (SD) for continuous variables and count and percentage for categorical variables. Due to data protection regulations, categories with fewer than five observations were combined or suppressed, and medians were not reported as they could approximate individual-level values. Mixed-effects models were used to assess longitudinal changes in routine laboratory measurements from three months prior to pregnancy to three months after pregnancy. The outcomes were platelet count (x10^9^/L), hemoglobin (mmol/L), hematocrit, and leukocyte count (x10^9^/L) and fixed effects included pregnancy period, comprising the period three months prior to pregnancy, each of the pregnancy months, the postpartum period (from delivery to 14 days after birth), and the period from postpartum to three months after pregnancy. In a secondary analysis, we investigated the association between platelet trajectories and preeclampsia in ET pregnancies. The outcome was platelet count, and fixed effects included pregnancy period (before pregnancy, first trimester, second trimester, third trimester, post-partum, after pregnancy), preeclampsia status and their interaction. In both models, a random intercept for each woman was included to account for repeated measurements. Estimated marginal means with 95% confidence intervals (CI) derived from the mixed-effects model were used for graphical presentation of longitudinal changes in the included measurements. All statistical analyses were performed using SAS version 9.4 (SAS Institute, Cary, NC, USA). A two-sided p-value < 0.05 was considered statistically significant.

### Approvals

This study was approved by the Department of Research and Innovation in the Capital Region of Denmark (P-2023–14524), the Research Support Center in Region Zealand (25/14357), and by Statistics Denmark (710440). Ethical Approval is not required under Danish law as this is a register-based study.

## Results

### Study population

In this study, we included 35 women with MPN, 30 women with ET, and 5 with PV. Baseline characteristics are summarized in Table 1. In women with ET, the mean age at MPN diagnosis was 30.1 years, the mean time from diagnosis to pregnancy was 50.1 months, and the mean year of diagnosis was 2013. None of the women with ET had pre-existing hypertension, while 17% had pre-existing diabetes. In women with PV, mean age at MPN diagnosis was 34.1 years, mean time from diagnosis to pregnancy was 13.8 months, and mean year of diagnosis was 2019. None of the PV women had pre-existing hypertension or diabetes.

**Table 1:** Baseline characteristics of women with MPN. Data is described by mean (SD) for continuous variables and n (%) for categorical variables. ET: essential thrombocythemia, MPN: myeloproliferative neoplasm, PV: polycythemia vera, SD: standard deviation

	ET	PV
*n*	30	5
Age at diagnosis (years)	30.1 (4.8)	34.1 (5.5)
Diagnosis to pregnancy (months)	50.1 (48.4)	13.8 (9.8)
Year of diagnosis	2013 (5.3)	2019 (1.6)
Pre-existing diabetes	5 (17)	0 (0)
Pre-existing hypertension	0 (0)	0 (0)

### Pregnancy characteristics

A total of 46 singleton pregnancies were included, and pregnancy characteristics stratified by MPN entity are summarized in Table 2. We included 41 pregnancies in 30 women with ET. Mean maternal age at pregnancy was 34.8 years, mean BMI was 24.8 kg/m^2^, and < 5 women were smoking during pregnancy. Most ET women delivered vaginally, with 37% delivering spontaneous and 29% induced, while 34% had a C-section. The mean gestational age at delivery was 39.1 weeks, the mean birth weight 3429 g, the birth weight z-score 0.27, the mean placental weight 608 g, the fetal-to-placental weight-ratio 5.56, and 39% of newborn were female gender. In ET pregnancies, 29% had maternal complications, including gestational hypertension, preeclampsia, gestational diabetes mellitus, intrahepatic cholestasis of pregnancy and thromboembolic events, and 27% had fetal complications, including preterm birth, small-for-gestational-age and large-for-gestational-age. The most common maternal complication was preeclampsia, affecting 12% of all ET pregnancies, while the most common fetal complication was large-for-gestational-age, affecting 12% of newborns. No stillbirths were reported. Due to data protection regulations, we could not report pregnancy characteristics in ET pregnancies stratified by preeclampsia status. However, gestational age at delivery, birth weight, birth weight z-score (no: 0.16 vs. preeclampsia: −0.30), placental weight, and fetal-to-placental weight-ratio (no: 5.88 vs. preeclampsia: 4.35) were lower in preeclampsia pregnancies.

**Table 2:** Platelet, hemoglobin, hematocrit and leukocyte trajectories in ET and PV pregnancies. Estimated mean levels of hematological parameters with 95% CI. CI: confidence intervals. ET: essential thrombocythemia, PV: polycythemia vera

	ET	PV
*n*_women_	30	5
n _pregnancies_	41	5
Maternal age (years)	34.8 (4.9)	35.2 (5.6)
Maternal BMI (kg/m ^2^)	24.8 (5.0)	24.1 (5.5)
Smoking during pregnancy	< 5	0 (0)
Mode of delivery		
***Spontaneous vaginal***	15 (37)	< 5
***Induced vaginal***	12 (29)	< 5
***C-section***	14 (34)	< 5
Gestational age at delivery (weeks)	39.1 (2.2)	40.1 (1.7)
Birth weight (g)	3429 (648)	3454 (673)
Birth weight z-score	0.27 (1.03)	−0.03 (1.14)
Placental weight (g)	608 (121)	629 (108)
Fetal-to-placental weigth-ratio	5.56 (0.93)	5.56 (0.63)
Female gender	16 (39)	< 5
Maternal complications	12 (29)	0 (0)
***Gestational hypertension***	*< 5*	*-*
***Preeclampsia***	*5 (12)*	*-*
***Gestational diabetes***	*< 5*	*-*
***Intrahepatic cholestasis of pregnancy***	*< 5*	*-*
***Thromboembolic event***	*< 5*	*-*
Fetal complications	11 (27)	0 (0)
***Preterm birth < 37 weeks***	*< 5*	*-*
***Small-for-gestational-age***	*< 5*	*-*
***Large-for-gestational-ale***	*5 (12)*	*-*

We included 5 pregnancies in 5 women with PV. Due to data protection regulations, we could not report categorical variables for PV pregnancies. Mean maternal age at pregnancy was 35.2 years, mean BMI was 24.1 kg/m^2^, and none of the women were smoking during pregnancy. The mean gestational age at delivery was 40.1 weeks, the mean birth weight was 3454 g, the birth weight z-score was − 0.03, the mean placental weight was 629 g, and the fetal-to-placental weight-ratio was 5.56. None of the PV pregnancies were complicated by any maternal or fetal complications, and no stillbirths were reported.

### Hematologic trajectories during pregnancy in women with ET and PV

**Fig. 2:**
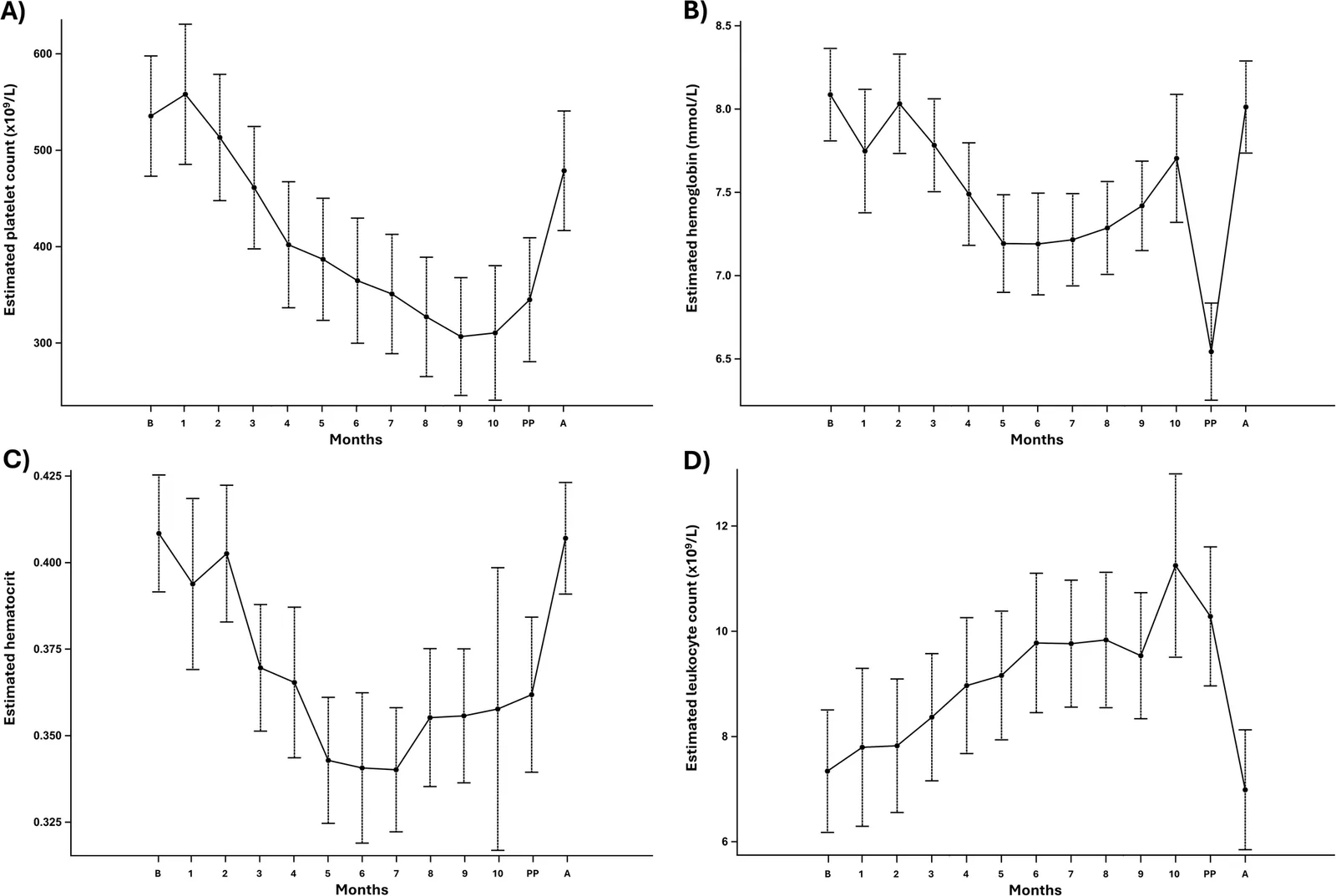
Estimated mean levels of hematological parameters with 95% confidence intervals by months (3 months prior to pregnancy to 3 months after pregnancy) in essential thrombocythemia pregnancies from the mixed-effects model. Error bars represent 95% confidence intervals. B: Before pregnancy (three months prior to pregnancy to conceiving), PP: postpartum (14 days after birth), A: after pregnancy (from postpartum to three months after pregnancy). (**A**) Estimated mean platelet count (x 10^9^/L). (**B**) Estimated mean hemoglobin level (mmol/L). (**C**) Estimated mean hematocrit. (**D**) Estimated mean leukocyte count (x 10^9^/L)

**Fig. 3:**
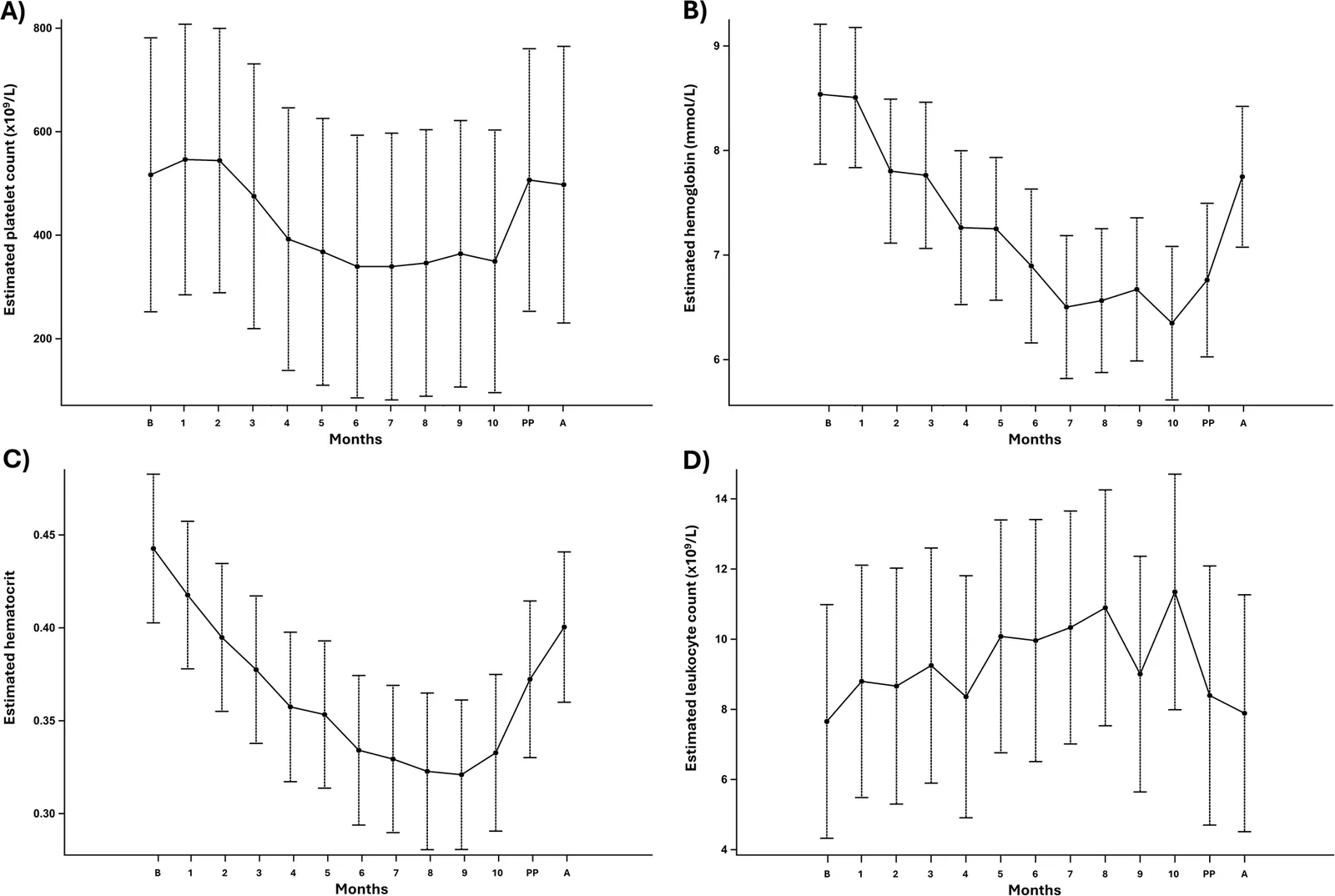
Estimated mean levels of hematological parameters with 95% confidence intervals by months (3 months prior to pregnancy to 3 months after pregnancy) in polycythemia vera pregnancies from the mixed-effects model. Error bars represent 95% confidence intervals. B: Before pregnancy (three months prior to pregnancy to conceiving), PP: postpartum (14 days after birth), A: after pregnancy (from postpartum to three months after pregnancy). (**A**) Estimated mean platelet count (x 10^9^/L). (**B**) Estimated mean hemoglobin level (mmol/L). (**C**) Estimated mean hematocrit. (**D**) Estimated mean leukocyte count (x 10^9^/L)

We investigated hematologic trajectories in ET and PV pregnancies using mixed-effects models. All women had multiple measurements during the study period, with a total of 15–78 measurements per month. Platelet count and hemoglobin were the most frequent analyses. Longitudinal trajectories of platelet, hemoglobin, hematocrit and leukocyte count trajectories in ET and PV pregnancies are illustrated in Figs. 2A-D and 3A-D, respectively. Estimated mean levels at baseline, the first month of pregnancy, delivery, postpartum and after pregnancy are presented in Table 3.

**Table 3:** Platelet, hemoglobin, hematocrit and leukocyte trajectories in ET and PV pregnancies. Estimated mean levels of hematological parameters with 95% CI. CI: confidence intervals. ET: essential thrombocythemia, PV: polycythemia vera

	ET	PV
	Mean (95% CI)	Mean (95% CI)
Platelet count (x 10^9^/L)		
Baseline	535 (473, 598)	517 (252, 781)
First month of pregnancy	558 (485, 631)	546 (285, 808)
Delivery	311 (241, 380)	350 (96, 603)
Postpartum	345 (281, 409)	507 (253, 760)
After pregnancy	479 (417, 541)	498 (231, 765)
Hemoglobin (mmol/L)		
Baseline	8.1 (7.8, 8.4)	8.5 (7.9, 9.2)
First month of pregnancy	7.7 (7.4, 8.1)	8.5 (7.8, 9.2)
Delivery	7.7 (7.3, 8.1)	6.3 (5.6, 7.1)
Postpartum	6.5 (6.2, 6.8)	6.7 (6.0, 7.5)
After pregnancy	8.0 (7.7, 8.2)	7.7 (7.1, 8.4)
Hematocrit		
Baseline	0.41 (0.39, 0.43)	0.44 (0.40, 0.48)
First month of pregnancy	0.39 (0.37, 0.42)	0.42 (0.38, 0.46)
Delivery	0.36 (0.31, 0.40)	0.33 (0.29, 0.37)
Postpartum	0.36 (0.34, 0.38)	0.37 (0.33, 0.42)
After pregnancy	0.41 (0.39, 0.42)	0.40 (0.36, 0.44)
Leukocyte count (x 10 ^9^ /L)		
Baseline	7.3 (6.2, 8.5)	7.7 (4.3, 11.0)
First month of pregnancy	7.8 (6.3, 9.3)	8.8 (5.5, 12.1)
Delivery	11.3 (9.5, 13.0)	11.3 (8.0, 14.7)
Postpartum	10.3 (9.0, 11.6)	8.4 (4.7, 12.1)
After pregnancy	7.0 (5.8, 8.1)	7.9 (4.5, 11.3)

In ET pregnancies, platelet counts (x 10^9^/L) declined significantly from baseline to delivery (−225; 95% CI −275, −175; *p* < 0.001), postpartum (−190; 95% CI −232, −149; *p* < 0.001), and after pregnancy (−57; 95% CI −94, −20; *p* = 0.003) (Fig. 2A). Hemoglobin levels (mmol/L) declined significantly from baseline to the first month of pregnancy (−0.3; 95% CI −0.7, 0.0; *p* = 0.05), delivery (−0.4; 95% CI −0.7, 0.0; *p* = 0.04), and postpartum (−1.5; 95% CI −1.8, −1.3; *p* < 0.001) (Fig. 2B). Hematocrit declined significantly from baseline to delivery (−0.05; 95% CI −0.09, −0.01; *p* = 0.009), and postpartum (−0.05; 95% CI −0.07, −0.02; *p* < 0.001) (Fig. 2C). Leukocyte counts (x 10^9^/L) increased significantly from baseline to delivery (3.9; 95% CI 2.3, 5.5; *p* < 0.001), and postpartum (2.9; 95% CI 1.8, 4.1; *p* < 0.001) (Fig. 2D).

In PV pregnancies, platelet counts declined significantly from baseline to delivery (−167; 95% CI −283, −51; *p* = 0.006) (Fig. 3A). Hemoglobin levels declined significantly from baseline to delivery (−2.2; 95% CI −2.8, −1.6; *p* < 0.001), postpartum (−1.8; 95% CI −2.4, −1.2; *p* < 0.001), and after pregnancy (−0.7; 95% CI −1.2, −0.4; *p* < 0.001) (Fig. 3B). Hematocrit declined significantly from baseline to the first month of pregnancy (−0.03; −0.05, 0.00; *p* = 0.02), delivery (−0.11; −0.14, −0.08; *p* < 0.001), postpartum (−0.07; 95% CI −0.10, −0.04; *p* < 0.001), and after pregnancy (−0.04; −0.06, −0.02; *p* < 0.001) (Fig. 3C). Leukocyte counts increased significantly from baseline to delivery (3.7; 95% CI 1.5, 5.9; *p* = 0.002) (Fig. 3D).

### Platelet count trajectories and preeclampsia in ET pregnancies

**Fig. 4:**
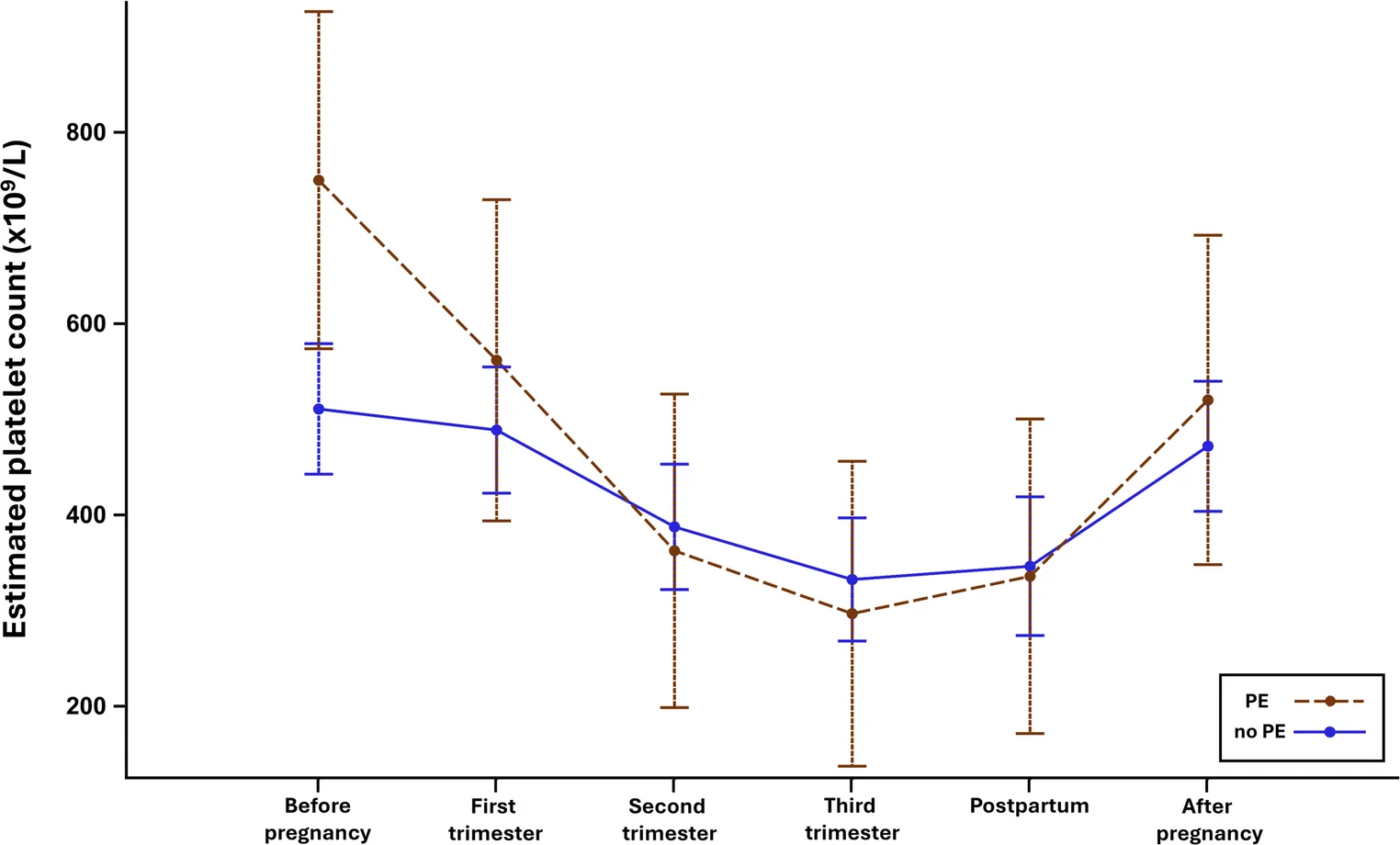
Estimated mean platelet counts with 95% confidence intervals by pregnancy period (before pregnancy, first trimester, second trimester, third trimester, post-partum, after pregnancy) and PE status in essential thrombocythemia pregnancies from the mixed-effects model. Error bars represent 95% confidence intervals. *PE* preeclampsia

In a secondary analysis, we used mixed-effect models to assess platelet trajectories (x 10^9^/L) in ET women stratified by preeclampsia status (Fig. 4). The estimated mean platelet counts are presented in Table 4. Platelet count trajectories differed significantly according to preeclampsia status (interaction F-value 35, *P* < 0.001). In women without preeclampsia, we observed a significant decline in platelet counts from first to second trimester (−101; 95% CI −129, −73; *p* < 0.001), and second to third trimester (−55; 95% CI −79, −31; *p* < 0.001), and a significant increase from postpartum to after pregnancy (125; 95% CI 80, 171; *p* < 0.001). In women with preeclampsia, platelet counts declined significantly from baseline to first trimester (−188; 95% CI −293, −84; *p* < 0.001), first to second trimester (−199; 95% CI −279, −119; *p* < 0.001), and second to third trimester (−66; 95% CI −124, −8; *p* = 0.03), and a significant increase from postpartum to after pregnancy (184; 95% CI 95, 273; *p* < 0.001) (Fig. 4). We observed a significant difference in platelet count between women without and with preeclampsia in the first trimester (−239; 95% CI −428, −50; *p* = 0.01) at baseline (Table 4).

**Table 4:** Platelet trajectories in ET pregnancies stratified by preeclampsia status. Estimated mean platelet counts and mean differences between women without and with PE with 95% CI. CI: confidence intervals

	No preeclampsia	Preeclampsia		
	Mean (95% CI)	Mean (95% CI)	Mean difference (95% CI)	*p*-value
Platelet count (x 10^9^/L)				
Baseline	511 (443, 579)	750 (574, 927)	−239 (−428, −50)	0.01
First trimester	489 (423, 555)	562 (394, 730)	−73 (−253, 107)	0.4
Second trimester	388 (322, 453)	363 (199, 527)	25 (−151, 202)	0.8
Third trimester	333 (268, 397)	297 (137, 456)	36 (−136, 208)	0.7
Postpartum	347 (274, 419)	336 (172, 500)	11 (−169, 190)	> 0.9
After pregnancy	472 (404, 540)	520 (348, 693)	−48 (−234, 137)	0.6

## Discussion

In this study, we characterized pregnancy outcomes and investigated hematologic trajectories in ET and PV pregnancies. None of the PV pregnancies were affected by maternal or fetal complications, while 29% of ET pregnancies were affected by maternal complications, and 27% of fetal complications. The most common fetal complication was large-for-gestational-age (12%), and the most common maternal complication was preeclampsia (12%). We observed a significant decline in platelet counts, hemoglobin levels, and hematocrit from baseline to delivery in both ET and PV pregnancies, whereas leukocyte counts increased. In a secondary analysis, women with ET who developed preeclampsia had significantly higher pre-pregnancy platelet counts and displayed a greater decline in platelet counts during pregnancy than women without preeclampsia. Together, these observations suggest that hematologic trajectories could aid in pregnancy risk stratification among women with ET.

In normal pregnancy, platelet counts decline by approximately 17%, primarily due to plasma volume expansion and storage of platelets in the spleen and placenta [13, 14]. We observed that platelet counts declined by 42% from baseline to delivery and returned to 87% of baseline after pregnancy in women with ET, whereas in PV the decline was 32% with recovery to 97% of baseline after pregnancy. Our findings are consistent with previous studies reporting a 43–48% decline in platelet counts from baseline to delivery in ET pregnancies with approximately 75% recovery after pregnancy [10, 15]. Thus, the observed platelet count trajectories are consistent with normal pregnancy physiology but appear more pronounced in ET and PV pregnancies. Hemoglobin levels and hematocrit are also expected to decline during pregnancy, whereas leukocyte counts increase substantially [16]. In line with this, we observed a modest decline in hemoglobin (−5%) and hematocrit (−12%) and a significant increase in leukocyte counts (+ 55%) in ET pregnancies. These findings are within the expected range during pregnancy and comparable to a previous study reporting a 9% decline in hemoglobin and a 36% increase in leukocyte counts from baseline to delivery in women with ET [10, 16]. Hemoglobin dropped approximately 1 mmol/L from delivery to postpartum in ET pregnancies, compatible with a peripartum blood loss of approximately 500–600 mL [17]. In contrast, hemoglobin levels (−25%) and hematocrit (−26%) declined substantially in PV pregnancies, exceeding the expected physiological ranges, whereas leukocyte counts increased (+ 47%) within the expected range. This suggests that hemoglobin trajectories in ET largely reflect physiological hemodilution, whereas the more pronounced decline in PV likely reflects entity-related factors, including higher baseline values and potential effects of clinical management during pregnancy, such as phlebotomy and, in some cases, interferon treatment.

We observed a high proportion of pregnancy complications in ET pregnancies, which is in line with previous studies. Maternal complications occurred in 29% of ET pregnancies, with preeclampsia being the most frequent (12%). Preeclampsia is reported to be the most common maternal pregnancy complication [18, 19], even though a recent study by Landtblom et al. reported that the incidence of preeclampsia among MPN women were comparable with the general population [5]. Notably, the prevalence of preeclampsia in our cohort (12%) was higher than that observed in the Danish general population (2.8–3.0%) [20]. In ET pregnancies, platelet trajectories differed between women with and without preeclampsia. Women who developed preeclampsia had significantly higher platelet counts before pregnancy and showed a 60% decline from baseline to third trimester, compared with a 32% decline in women without preeclampsia. Notably, women with preeclampsia already showed a significant decline in the first trimester, which was not observed in women without preeclampsia. These findings are compatible with the pathophysiology of preeclampsia, in which elevated platelet counts in early pregnancy might contribute to impaired placentation, subsequent endothelial dysfunction, and fetal growth restriction [21]. The pronounced platelet decline may reflect early platelet consumption driven by endothelial dysfunction. Further supporting this notion, pregnancies complicated by preeclampsia were also associated with lower birthweight, birthweight z‑scores, placental weight, and a lower fetal‑to‑placental weight ratio compared with non‑preeclampsia pregnancies. Together, these findings are consistent with placental dysfunction and impaired fetal growth, even in the absence of the classical small-for-gestational-age classification. Contrary to our findings, How et al. observed that a more pronounced decline in platelet counts during pregnancy was associated with a decreased risk of pregnancy complications [10]. Unlike How et al., our cohort only included pregnancies resulting in a registered birth in the MBR, thereby not including fetal losses before gestational week 22 [10]. A fetal loss would naturally be associated with a smaller decline in platelet counts, as the physiological adaptation to pregnancy is interrupted. Thus, a more pronounced decline in platelet counts during pregnancy might be associated with a reduced risk of fetal loss or thromboembolic events, but not necessarily to a lower risk of preeclampsia. Although based on a small number of women, the association between platelet count trajectories and preeclampsia suggests that platelet counts could have potential as an early risk marker for preeclampsia in ET pregnancies. Moreover, the association between higher pre-pregnancy platelet counts and subsequent preeclampsia suggests that pre-pregnancy disease control and platelet count normalization may be important in women with ET. However, these exploratory findings are based on a small number of preeclampsia cases and require validation in larger cohorts before specific clinical recommendations can be made.

Previous studies have reported high rates of fetal complications in MPN pregnancies with preterm birth and small-for-gestational-age being the most common [5, 7, 18]. In our study, fetal complications occurred in 27% of ET pregnancies. Due to Danish data protection regulations, counts for small-for-gestational-age and preterm birth could not be reported, however, 12% of newborns were classified as large-for-gestational-age (> 95th percentile). Large-for-gestational-age is not a commonly evaluated outcome in the existing literature on MPN pregnancies, and only a few studies have reported this outcome. Alimam et al. reported that 8% of newborns of MPN mothers were > 95th percentile, which is in line with our findings [7]. One explanation could be that large-for-gestational-age is driven by the higher prevalence of diabetes. We observed that 17% of ET women had pre-existing diabetes, which is higher than expected. Gangat et al. found that 4% of pregnant women with ET had diabetes, which was strongly associated with both increased risk of first‑trimester pregnancy loss (67% vs. 23%) and a higher incidence of preeclampsia (33% vs. 5%) [15]. However, the observed association between diabetes and MPN might reflect detection bias, as women with diabetes undergo more frequent healthcare contacts and laboratory testing, potentially increasing the likelihood of diagnosing an otherwise asymptomatic MPN. Nevertheless, diabetes may contribute to large-for-gestational-age births, in contrast to the restricted fetal growth often reported in MPN pregnancies.

In our study, the gender distribution among newborns (61% male) deviated from the expected distribution. Although this might be due to random variation given the small sample size, it could reflect shifts in the overall inflammatory balance, influencing sex‑specific embryo survival. Arkema et al. found that women with systemic lupus erythematosus or rheumatoid arthritis had a slightly reduced proportion of male births, likely reflecting inflammation‑related early loss of male embryos [22]. Radin et al. found that preconception low‑dose aspirin increased the proportion of male live births, particularly among women with higher systemic inflammation, suggesting that aspirin may counteract inflammation that would otherwise reduce survival of male embryos [23]. These observations suggest that the inflammatory environment and anti‑inflammatory treatments, including aspirin, may influence fetal sex ratios in MPN pregnancies, but further studies are needed.

The strengths of the study include the longitudinal laboratory data from regional information systems and the linkage to Danish health registries with no loss to follow-up. Limitations include the small sample size, especially of PV pregnancies. Additionally, we lacked detailed information on disease activity, treatment regimens, including interferon use before and during pregnancy, and *JAK2/CALR/MPL* mutation status, all of which may influence hematologic patterns and the risk of pregnancy complications. While the majority of women with PV and approximately half of those with ET are expected to carry the *JAK2V617F* mutation, with most of the remaining ET cases being CALR-mutated, the lack of individual-level mutation data limits subgroup analyses [1]. Another aspect of the study is that hematologic measurements were based on routine clinical testing. However, women with MPN are monitored closely during pregnancy, and all included women had frequent measurements throughout pregnancy. Thus, the laboratory data reflect real-world clinical practice.

In conclusion, hematological trajectories during pregnancy largely follow normal physiological patterns with declining platelet counts, hemoglobin, and hematocrit from baseline to delivery, and increasing leukocyte counts. However, platelet declines are more pronounced in ET pregnancies, and the decline of hemoglobin and hematocrit in PV pregnancies exceeds expected physiological ranges. Women who developed preeclampsia had significantly higher platelet counts before pregnancy and showed a greater decline towards delivery compared with women without preeclampsia, suggesting that platelet trajectories might be associated with risk of preeclampsia. These findings highlight the need for close hematologic surveillance during pregnancy and further research into early predictors of pregnancy complications in MPN.

## Data Availability

The data used in this study are not publicly available due to national data protection regulations. Access to aggregated data may be granted upon reasonable request and approval from the relevant authorities.
